# Exercise and Nutrition Interventions for Prehabilitation in Hepato-Pancreato-Biliary Cancers: A Narrative Review

**DOI:** 10.3390/nu15245044

**Published:** 2023-12-08

**Authors:** Cami N. Christopher, Dong-Woo Kang, Rebekah L. Wilson, Paola Gonzalo-Encabo, Salvatore Ficarra, Diane Heislein, Christina M. Dieli-Conwright

**Affiliations:** 1 Department of Medical Oncology, Division of Population Health Sciences, Dana-Farber Cancer Institute, Boston, MA 02215, USA; cameron_christopher@dfci.harvard.edu (C.N.C.); dong-woo_kang@dfci.harvard.edu (D.-W.K.); rebekahl_wilson@dfci.harvard.edu (R.L.W.); paola_gonzaloencabo@dfci.harvard.edu (P.G.-E.); salvatore.ficarra03@unipa.it (S.F.); 2 Department of Nutrition, Harvard T.H. Chan School of Public Health, Boston, MA 02115, USA; 3 Harvard Medical School, Boston, MA 02115, USA; 4 Departamento de Ciencias Biomédicas, Área de Educación Física y Deportiva, Facultad de Medicina y Ciencias de la Salud, Universidad de Alcalá, 28801 Madrid, Spain; 5 Sport and Exercise Sciences Research Unit, Department of Psychology, Educational Science and Human Movement, University of Palermo, 90133 Palermo, Italy; 6 Department of Physical Therapy, Sargent College of Health and Rehabilitation Sciences, Boston University, Boston, MA 02215, USA

**Keywords:** physical activity, exercise, nutrition, prehabilitation, hepato-pancreato-biliary cancer, liver cancer, pancreatic cancer, biliary cancer, cancer survivors

## Abstract

Gastrointestinal (GI) cancers constitute over 25% of global cancer cases annually, with hepato-pancreato-biliary (HPB) cancers presenting particularly poor prognosis and challenging surgical treatments. While advancements in clinical care have improved post-operative outcomes over time, surgery for HPB cancers remains associated with high morbidity and mortality rates. Patients with HPB cancer are often older, diagnosed at later stages, and have a higher prevalence of co-morbid conditions, leading to reduced life expectancy, suboptimal post-operative recovery, and increased recurrence risk. Exercise and nutrition interventions have emerged as safe non-pharmacological strategies to enhance clinical outcomes among cancer survivors, but their potential in the pre-operative period for patients with HPB cancer remains underexplored. This narrative review evaluates existing evidence on exercise and nutritional interventions during pre-operative prehabilitation for HPB cancer populations, focusing on clinically relevant post-operative outcomes related to frailty and malnutrition. We conducted a literature search in PubMed and Google Scholar databases to identify studies utilizing a prehabilitation intervention in HPB cancer populations with exercise and nutritional components. The currently available evidence suggests that incorporating exercise and nutrition into prehabilitation programs offers a critical opportunity to enhance post-operative outcomes, mitigate the risk of comorbidities, and support overall survivorship among HPB cancer populations. This review underscores the need for further research to optimize the timing, duration, and components of pre-operative prehabilitation programs, emphasizing patient-centered, multidisciplinary approaches in this evolving field.

## 1. Introduction

Gastrointestinal (GI) cancers are a growing public health burden, with a significant impact on both morbidity and mortality. In 2018, the global incidence estimated 4.8 million new cases of GI cancers, including those of the stomach, liver, esophagus, pancreas, and colorectum, and accounted for over 26% of cancer cases and 35.4% of cancer deaths [1,2]. Alarming projections suggest that the incidence and mortality rates of GI cancers, particularly hepato-pancreato-biliary (HPB) cancers encompassing the liver, gallbladder, intrahepatic bile duct, and pancreas malignancies, could increase by 24–45% by 2040 [1,3,4].

The rising burden of HPB cancers underscores the urgent need for not only preventive measures but also enhanced clinical services to improve the care of patients and survivors undergoing treatment for these cancers. The prognosis of HPB cancers tends to be poor, with liver and pancreatic cancers exhibiting some of the lowest 5-year survival rates [5]. Moreover, treatment modalities for HPB cancers including surgery and chemotherapy pose formidable challenges, such as an increased risk of frailty, morbidity, and mortality [1,5,6,7,8,9,10].

Surgical resection, encompassing procedures like hepatic resection and transplantation, stands as one of the primary treatment options for HPB malignancies, though this is largely dependent on the stage of cancer and resectability of the tumor at time of detection [11,12,13,14,15]. While advancements in clinical practice and technology have led to improved post-operative outcomes over time, surgical intervention for HPB cancers remains associated with elevated rates of post-operative morbidity and mortality [1,4,6,7,9,11,12,13,14,16,17]. Patients diagnosed with HPB cancers tend to be older and exhibit a higher prevalence of co-morbid conditions such as sarcopenia, malnutrition, and liver cirrhosis [18,19,20,21]. These risk factors can contribute to reduced life expectancy and poorer surgical outcomes among HPB cancer populations.

Notably, pre-operative risk factors, including frailty and malnutrition, have emerged as crucial predictors of post-operative complications and overall survival among patients with HPB cancer [1,17,21,22,23,24,25,26]. Frailty is a condition characterized by increased vulnerability due to age-related physiological decline in physical, functional, and cognitive capacities, and it encompasses features like exhaustion, low fitness levels, impaired physical function, and changes in body composition [20,27,28,29]. The symptoms of frailty can result in unintentional weight loss, declines in muscle and bone mass, and development of sarcopenia and malnutrition [20,27,28,29]. Malnutrition, a component of frailty, involves nutrient imbalances and decreases in fat-free mass [30,31]. Among cancer populations, malnutrition can lead to cachexia, which has adverse effects including the loss of lean body mass, increased muscle wasting, impaired physical function, declines in mental function and health-related quality of life, and poorer clinical survival outcomes [30,31,32].

The potential promise of prehabilitation interventions emerges as a means to address pre-operative frailty and malnutrition-related risk factors and to enhance survivorship outcomes in patients and survivors with HPB cancer [10,33,34,35]. Prehabilitation interventions are multi-disciplinary approaches that leverage the pre-operative period to mitigate surgery-related functional decline and its consequences. These interventions typically encompass exercise training, nutritional therapy, smoking cessation, and psychosocial support (e.g., stress and anxiety reduction and social support) [26,35,36,37,38,39].

While exercise during the prehabilitation period has demonstrated its safety and feasibility in improving physical, nutritional, and psychosocial outcomes among patients with cancer [26,40,41], it remains unclear whether prehabilitation programs can yield benefits beyond mitigating post-operative complications in HPB cancer populations [33,34,42,43,44]. Due to the complexity of HPB cancers, only a limited number of studies have explored pre-operative prehabilitation interventions, specifically focusing on exercise and nutrition components and their impact on survivorship [33,34,42,43,44].

Therefore, the primary objective of this review is to synthesize the existing literature on pre-operative prehabilitation interventions that incorporate exercise, with or without a nutrition component, in HPB cancer populations. This review aims to comprehensively describe the intervention strategies with respect to clinically relevant outcomes associated with frailty and malnutrition, encompassing post-operative complications, fitness levels, strength, physical function, body composition, and biomarkers of nutritional status. By doing so, we aim to provide valuable insights to better prepare patients with HPB cancer for surgery and enhance their prospects for survivorship.

## 2. Methods

PubMed, Google Scholar, ClinicalTrials.gov, and NIH reporter were, respectively, searched for published and ongoing studies published through 31 August 2023. The search strategy included combinations of the following terms: hepato-pancreato-biliary cancer, hepatobiliary cancer, liver cancer, hepatocellular carcinoma, pancreatic cancer, bile duct or biliary cancer, pre-operation, prehabilitation, physical activity, exercise, nutrition, dietary intervention, oral nutritional supplements, physical function, physical fitness, frailty, malnutrition, sarcopenia, and survival. The key criterion was to identify relevant trials with pre-operative or peri-operative interventions including an exercise component, or both exercise and nutritional intervention components among HPB cancer populations with a measured outcome relating to frailty or malnutrition (e.g., post-operative complications, fitness levels, strength, physical function, body composition, and biomarkers of nutritional status). Trials that only had a post-operative intervention, did not have relevant intervention components, or were not targeting patients with HPB cancer were not included in this narrative review. Randomized clinical trials (RCTs), single-group studies, and non-randomized trials were prioritized, and we excluded case reports, editorials, protocol papers, and commentaries.

## 3. Design of Prehabilitation Programs in HPB Cancer Populations

This review considered twelve studies (across 13 publications), focusing on pre-operative and peri-operative prehabilitation interventions in HPB cancer populations, with an emphasis on exercise-only or exercise and nutrition multimodal approaches (Table 1 and Table 2). The studies primarily targeted pancreatic cancer [45,46,47,48,49,50,51,52], hepatocellular carcinoma [52,53], and various combinations of HPB malignancies [54,55,56,57,58]. Regarding the treatment status of participants, all exercise-only prehabilitation interventions included patients with HPB cancer undergoing treatments during the prehabilitation period, including mixed therapies [45,46,54] or neoadjuvant chemotherapy alone or with radiation [47,48,49,50]. Among the multimodal studies, only four reported the participants’ treatment status throughout the prehabilitation program, with two studies indicating no additional treatment during prehabilitation [51,56], and two studies noting patients undergoing neoadjuvant chemotherapy [55,58].

The reviewed studies encompassed four exercise-only prehabilitation programs (across seven publications) [45,46,47,48,49,50,54], and seven multimodal interventions [51,52,53,55,56,57,58]. The duration of these interventions varied, ranging from a minimum of 1 week to up to 16 weeks. The variation in intervention duration was influenced by the pre-operative prehabilitation period and whether patients received concurrent treatments, such as neoadjuvant chemotherapy and/or radiation.

Exercise-only prehabilitation studies predominantly prescribed combined (COMB) exercise interventions, incorporating both aerobic exercise (AE) and resistance exercise (RE). The AE component primarily involved walking or the use of AE machines (e.g., ergometers, treadmills, or stair steppers), while the RE component utilized resistance bands, dumbbells, strength-training machines, and bodyweight exercises, as described further in Table 1 and Table 2.

Multimodal interventions included those with either AE-only or COMB approaches for the exercise component and a nutrition component. The nutritional component of multimodal interventions varied across studies, including protein supplementation and tailored nutritional support (e.g., dietary advice and consultations with nutritionists). In addition to exercise and nutritional components, three of the reviewed studies integrated psychological/psychosocial support and breathing exercises into their interventions (Table 1 and Table 2).

## 4. Impact of Prehabilitation on Frailty and Malnutrition-Related Outcomes in HPB Cancer Populations

### 4.1. Fitness, Strength, and Physical Function

Prehabilitation interventions have demonstrated potential in improving frailty-related outcomes, particularly in regard to cardiovascular fitness, strength, and physical function measures (Table 3). Across studies assessing the impact of prehabilitation on cardiovascular fitness and aerobic endurance, significant improvements in VO2_peak_ were consistently observed compared to pre-intervention levels and standard care groups [46,54,56]. These improvements were also evident in peak power output, another measure of cardiovascular fitness. However, other fitness measures such as muscular strength, heart rate reserve, and heart rate variability did not exhibit significant improvements with prehabilitation. It is important to note the limited number of prehabilitation studies conducted in HPB cancer populations, and the variability in measures used may contribute to these mixed results. Additionally, the studies included in this review have small sample sizes and/or lack a control group, which limits the interpretation and generalizability of the findings.

Concerning physical function outcomes, the evidence is less conclusive. While improvements in the 6 min walk test distance were generally observed across studies, findings regarding gait speed and grip strength were mixed (Table 3). Baimas-George et al. (2020) was the only study to explore measures of frailty-related indicators, reporting significant reductions in frailty phenotype, weight loss, and exhaustion, following the prehabilitation program [55]. However, the diverse measures of physical function across studies and the variations in measurement methodologies pose challenges in drawing definitive conclusions. While prehabilitation interventions appear beneficial for cardiovascular fitness and physical function, further research is needed to confirm these findings and determine the optimal exercise components in prehabilitation programs.

### 4.2. Biomarkers and Nutritional Status

To assess the impact of prehabilitation on pre-operative risk factors associated with malnutrition, including nutrient imbalances or inadequate nutrition, we examined biomarkers of nutritional status and nutrient imbalances (Table 4). Among studies that evaluated liver and renal function, prehabilitation maintained these biomarkers post-operation [53,55]. Metabolic function biomarkers, including glucose and insulin metabolism, did not show any significant differences following the prehabilitation interventions [53,55], except for in the study of Kaibori et al. (2019), who reported improved glucose and insulin metabolic biomarkers in the exercise and diet group at 3 months and 6 months post-operation compared to the diet-only control group [53].

Regarding nutritional status, most studies found that prehabilitation did not result in significant changes in nutritional status or serum albumin levels following the intervention. However, Nakijima et al. (2019) reported a significant increase in the prognostic nutritional index score and serum albumin levels following a 3-week multimodal prehabilitation program [56]. These findings suggest that prehabilitation programs generally do not worsen pre-operative risk factors related to biomarkers and nutritional status, but rather support maintenance through post-operation, with some studies even demonstrating potential improvements in risk factors of malnutrition.

### 4.3. Body Composition

We then explored the impact of prehabilitation on the clinical outcomes of body composition, including body weight, body mass index (BMI), skeletal muscle, and fat mass on outcomes of frailty and/or malnutrition. Studies assessing body weight and BMI reported either no significant changes or a significant decline, irrespective of exercise and diet components or usual care (Table 5). However, prehabilitation programs seemed to preserve or enhance skeletal muscle index or skeletal muscle mass. For example, Parker et al. (2021) found that prehabilitation exercise preserved skeletal muscle index compared to a significant decline observed in historical controls [49]. Similarly, Bui Ngoc et al. (2019) compared a multimodal prehabilitation program to a post-operation rehabilitation program and found that appendicular skeletal muscle index significantly decreased from pre-operation to 4 weeks post-operation in the rehabilitation group. However, it was maintained in the prehabilitation group [58]. Only Kaibori et al. (2019) examined fat mass and BMI, revealing greater fat loss around the waist in the combined exercise and diet intervention group compared to the diet-only group, with neither intervention affecting BMI [53].

The preservation of skeletal muscle is particularly relevant, as low skeletal muscle mass, weight loss, low BMI, and frailty are predictive of post-operative complications and poorer survival outcomes in patients with HPB cancer [1,17,21,22,23,24,25,26,59]. Although findings related to BMI and weight loss were limited and mixed, several factors, such as the composition of the prehabilitation program (exercise-only vs. exercise and nutrition), intervention duration, and adherence, may influence the effectiveness and feasibility of these programs in impacting body composition outcomes.

### 4.4. Post-Operative Outcomes

Regarding the clinical post-operative outcomes, the impact of prehabilitation varied across studies (Table 6). Some studies reported a reduced risk of post-operative complications, such as pulmonary complications, intra-operative blood loss, and transfusion, associated with prehabilitation [45,51,52]. However, other studies found no significant differences in post-operative complications between prehabilitation and control groups [54,55,58]. Similarly, prehabilitation was linked to a shorter hospital length of stay in certain studies, although this effect was not consistent across all studies.

In summary, while the reviewed studies differed in the clinical outcomes they assessed, a recurring trend of improved aerobic capacity was observed across studies. Despite varying effects on other post-operative and clinical outcomes, including fitness, physical function, nutritional biomarkers, body composition, and post-operative complications, pre-operative prehabilitation interventions combining exercise and nutrition components did not appear to exacerbate pre-operative risk factors or relevant clinical outcomes in HPB cancer populations. However, the heterogeneity of results, limited number of studies, small sample sizes, and lack of control group in some studies highlight the need for further research to provide more definitive conclusions on the effects of prehabilitation in this context.

## 5. Why Timing Matters

### 5.1. Alignment with Recent Guidelines

The implementation of pre-operative prehabilitation programs using multimodal interventions in patients with HPB cancer presents an opportunity to reduce pre-operative risk, optimize post-operative outcomes, and minimize the risk of long-term comorbidities associated with frailty and/or malnutrition [33,35,60]. The recently published Guidelines for Perioperative Care for Liver Surgery: Enhanced Recovery After Surgery (ERAS) Society Recommendations 2022 have underscored the importance of tailored prehabilitation programs lasting 4–6 weeks [35]. It is recommended that these programs encompass exercise, dietary interventions, and psychosocial components (e.g., anxiety reduction) for high-risk patients, including the elderly, malnourished individuals, overweight patients, and smokers, prior to liver surgery [35]. Notably, these guidelines strongly recommend nutritional assessment before all hepatic surgeries, advocating for enteral supplementation for malnourished patients at least 7–14 days prior to surgery [35].

### 5.2. Impact of Prehabilitation on High-Risk Patients

In line with the new guidelines, the findings of this review corroborate previous reviews emphasizing the importance of tailoring multimodal pre-operative interventions to high-risk patients with HPB cancers. Such tailored interventions have the potential to improve post-operative outcomes and effectively reduce the hospital length of stay in this patient population [33,42]. While our review revealed mixed results concerning the consistency of impact on post-operative clinical outcomes, pre-operative prehabilitation interventions have demonstrated their ability to address risk factors associated with impaired functional capacity and a loss of skeletal muscle. These interventions can help to mitigate frailty and prevent further malnutrition that may occur due to neoadjuvant treatments or the cancer itself [21,25,61,62]. Given the broad spectrum of post-operative outcomes that can be assessed and the significant clinical implications they bear on cancer survival, additional research is warranted to better understand the potential benefits of prehabilitation on clinically relevant post-operative outcomes in high-risk populations.

### 5.3. Challenges in Assessing Prehabilitation Outcomes

Previous studies have suggested a connection between higher levels of physical activity prior to abdominal surgical resection and a shorter time to functional recovery compared to insufficiently active individuals [17,63]. However, it is important to acknowledge that this association is not consistently observed across all studies. Alongside physical activity, nutritional interventions have the potential to enhance physical fitness and clinical outcomes during the pre-operative period [64]. Nevertheless, prehabilitation programs often vary in their approach, components included (e.g., exercise, nutrition, and psychosocial support), duration, frequency, and measurement methodologies, making it challenging to synthesize the impact of pre-operative interventions [38,55].

There is a pressing need for future research to continue exploring how multimodal prehabilitation interventions may improve frailty and malnutrition outcomes in patients with HPB cancer, as well as mitigate further deterioration and post-operative complications. It is crucial to emphasize that the window of opportunity for prehabilitation in this context is often brief, with time intervals typically ranging from 4 to 16 weeks and even shorter periods (e.g., 1–2 weeks) in some patients with HPB cancer [65]. In light of the suggestive evidence observed in this review, multimodal prehabilitation interventions targeting aspects of fitness, physical function, and nutrition should be considered to deter the common physiologic decline observed in HPB cancer populations.

## 6. Biologic Mechanisms of Prehabilitation Exercise and Nutrition Interventions

### 6.1. Exercise-Related Biological Mechanisms

As established in this review, prehabilitative exercise and nutritional interventions offer a potential strategy to optimize patients’ condition before surgery and improve various post-operative recovery and frailty and malnutrition-related outcomes, including physical function, fitness, body composition, and nutritional deterioration, as well as post-operative morbidity and mortality for patients with HPB cancer [34,42,56]. While the precise biological mechanisms underpinning exercise prehabilitation for patients with HPB cancer are not fully elucidated, several explanations have been suggested. These mechanisms encompass improved cardiovascular function, reduced inflammation, enhanced insulin sensitivity, mood improvement, pain reduction, and increased muscle mass and strength [59,66].

Poorly managed pre-operative risk factors coupled with the physiologic stress of surgery can exacerbate the risk of frailty and malnutrition, leading to systemic inflammation, weight loss, metabolic dysfunction, loss of muscle mass, and nutrient deficiencies, thereby impairing the overall quality of life [59,66,67,68]. Prehabilitation interventions comprising exercise and nutritional components therefore offer a unique window of opportunity to intervene in patients at risk of further decline.

Exercise during prehabilitation may exert positive effects on post-operative outcomes and recovery through various mechanisms. For instance, exercise may enhance wound healing by facilitating the mobilization of exercise-induced peripheral endothelial progenitor cells, which may contribute to reducing the length of hospitalization [69]. Beyond peripheral effects, exercise-induced growth factors, such as brain-derived neurotrophic factor and insulin-like growth factor (IGF)-1, could have neuroprotective and neurogenic effects, potentially preventing post-operative neurocognitive disorders and cognitive dysfunction [70]. Moreover, long-term exercise can induce an anti-inflammatory state in the body and enhance the humoral immune response, characterized by an increased number of regulatory T cells [71]. Therefore, exercise may help to restore local and systemic immune balance before surgery, mitigate the onset of a post-operative inflammatory phase, and reduce the risk of developing post-operative complications [59,72].

However, it is worth noting that the impact of exercise during prehabilitation on pre-operative risk factors related to frailty and malnutrition may vary in effect based on the mode of exercise training. Exercise within prehabilitation can support the preservation and enhancement of physiologic reserves and functional capacity, affecting both the cardiovascular and musculoskeletal systems [72]. Aerobic and endurance exercise training are thought to alter frailty phenotypes through enhancements in aerobic capacity and increased muscle mass, whereas resistance exercise is known to support improvements in skeletal muscle mass, function, strength, and body composition [28]. The benefits of aerobic and resistance exercise alone may have synergistic effects through a combined approach for outcomes related to frailty and malnutrition. This synergy becomes particularly crucial in patients with HPB cancer, where the compounding burden of poor pre-operative health, age-related effects, and the stress of cancer-related treatments further challenge physiological resilience [60,72].

### 6.2. Nutritional Status and Surgical Stress

The potential mechanisms related to nutritional status in patients with HPB cancer further emphasize the importance of prehabilitation interventions prior to surgery. Surgical procedures necessitate a robust pre-operative physiological reserve to cope with surgical stress and the heightened demand for nutritional substrates [61,72]. Surgical stress, characterized by the release of stress hormones and cytokines proportional to the extent of tissue damage, can trigger an inflammatory response [73].

Patients entering surgery with poor pre-operative nutritional status and low physiological reserves, such as those who are malnourished, frail, or sarcopenic, may rapidly deplete their nutritional reserves post-surgery. This depletion can negatively impact their ability to tolerate surgical stress, leading to poor post-operative outcomes, an increased risk of infections, and further declines in skeletal muscle mass and fitness [74,75]. Therefore, enhancing the nutritional status of HPB cancer populations before surgery emerges as a crucial aspect of oncological management, aimed at preventing or ameliorating further nutritional decline [72,76].

## 7. Additional Considerations of Prehabilitation in HPB Cancer Populations

While the inclusion of exercise and nutrition prehabilitation interventions within the care plan of patients with HPB cancer to support frailty and malnutrition outcomes is promising, there are several considerations when deciding who would benefit most and what the program should entail.

The majority of patients with HPB cancers are likely to present with various co-existing conditions, such as cachexia, malnourishment, diabetes, and sarcopenia [77]. The number and severity of these established conditions at diagnosis can help to identify patients at the highest risk of experiencing poor surgical outcomes, and, therefore, those who are likely to benefit most from prehabilitation. For instance, patients who are frail are more likely to benefit from prehabilitation given the association of frailty with post-operative complications [55,78]. Thus, assessing risk factors and metrics of frailty and physical function before cancer treatments and operations becomes essential in informing the components of prehabilitation and supporting overall survival [8,72,75]. The information on co-morbidities and current health status will dictate the components of prehabilitation based on best practices for the identified conditions. For instance, the use of resistance training to maintain or improve skeletal muscle mass and physical function is recommended for individuals with sarcopenia [79,80,81]. Additionally, the receipt of neoadjuvant therapy during the pre-operative period and the anticipated adverse effects should also be considered when identifying patients who would benefit most from a prehabilitation program, as the functional health and physiological reserve of a patient are likely to further deteriorate throughout neoadjuvant treatment.

Precision exercise and nutrition prescriptions are the recommended processes to ensure patients receive a tailored program that will be most effective for them [82,83,84,85]. Given that the timeline for a prehabilitation program is often short (e.g., 2–4 weeks if surgery is the first treatment choice, and 2–6 months if receiving neoadjuvant therapy), identifying the primary intention of prehabilitation for each patient and subsequently prioritizing the most needed component(s) is critical to prescribing the most effective program. For instance, a multimodal prehabilitation program may involve any or all of the following, subject to the primary intention of the program: (1) optimization of physical fitness through exercise; (2) prevention of a catabolic state and support of an anabolic state through nutrition; (3) psychological intervention to reduce stress and improve overall emotional well-being; and/or (4) correction of any modifiable risk factors or comorbidities, such as alcohol consumption cessation [86].

Regardless of the number of components selected to establish a tailored prehabilitation program, the process should always involve a multidisciplinary team and be conducted in consultation with the patient’s full medical care team. This collaborative approach ensures that prehabilitation interventions are aligned with the individual needs and goals of each patient, optimizing their chances for successful surgical outcomes and overall well-being.

It is worth noting that not all patients diagnosed with HPB cancers will receive surgical treatment as part of their cancer treatments, particularly those that are diagnosed at advanced stages [9]. While the option to engage in pre-operative prehabilitation may not be accessible to these patients that do not receive surgical treatment, the call to integrate prehabilitative exercise and nutrition within the care plan following cancer diagnosis, prior to any cancer treatment, to address the risks of frailty and malnutrition on survivorship is still applicable within these populations [34,35,42,44,61,84,87,88].

## 8. Ongoing Studies

While prehabilitation interventions in patients with HPB cancers hold promise for improving short-term post-operative and long-term survivorship outcomes, the current findings are limited by the relatively small number of studies investigating a wide range of clinically relevant outcomes. In the studies reviewed, the heterogeneous approaches to intervention design and outcomes assessed made it challenging to synthesize the potential benefits of exercise and nutrition interventions during pre-operative prehabilitation programs in patients with HPB cancer. However, ongoing and active research efforts are poised to address these limitations and expand our knowledge in this area (Table 7) [89,90,91,92,93,94,95,96,97,98,99,100,101,102,103].

Of the fifteen ongoing studies we identified, nine studies include multimodal prehabilitation interventions, incorporating both exercise and nutrition components (Table 7). These ongoing studies are investigating outcomes of interest that will provide valuable insights into the physiologic effects of prehabilitation interventions in HPB cancer populations. Importantly, all these studies have identified frailty or malnutrition-related outcomes as primary or secondary endpoints of interest. It is noteworthy that most ongoing studies have a particular emphasis on pancreatic cancer within the HPB cancer group, aligning with population trends observed in the reviewed studies. The impact of these ongoing trials will not only inform the feasibility and effectiveness of prehabilitation interventions on outcomes related to frailty and malnutrition across HPB cancer populations, but will also shed light on the specific challenges and opportunities associated with pancreatic cancer.

Several of the ongoing studies also include a follow-up component into a rehabilitative period, providing a unique opportunity to assess both short-term post-operative outcomes and longer-term survivorship outcomes. These studies are expected to contribute significantly to our understanding of the sustainability of prehabilitation effects and the potential for long-term improvements in functional capacity and quality of life for patients with HPB cancer. Ongoing research efforts in the field of prehabilitation for HPB cancer populations hold great promise for addressing the current gaps in knowledge, standardizing intervention approaches, and providing comprehensive insights into the benefits of prehabilitation in improving outcomes for these patients.

## 9. Future Directions

Given the escalating burden of HPB cancer and recommendations in recent guidelines, the inclusion of prehabilitation interventions, encompassing exercise and nutritional components, should be a consideration within clinical care. The findings of our narrative review suggest the potential significance of incorporating exercise and nutrition during prehabilitation in the continuum of cancer care. These interventions may offer a critical opportunity to enhance post-operative clinical outcomes, mitigate co-morbid risks, and bolster overall survivorship among patients with HPB cancer.

Healthcare providers and clinicians can consider the integration of exercise and nutrition prehabilitation programs into the comprehensive care plan for patients with HPB cancer. Tailoring these programs to individual patient needs, risk factors, and treatment plans can result in better overall health outcomes and improved quality of life. However, it is crucial to acknowledge that our understanding of prehabilitation in HPB cancer populations is still evolving. There remains a pressing need for further research in this area, with a focus on determining the optimal timing, duration, and components of prehabilitation programs. Additionally, ongoing research should explore the feasibility and effectiveness of extending prehabilitation into the post-operative and rehabilitative phases of care to ensure the long-term health and wellbeing of patients and survivors with HPB cancers.

## 10. Conclusions

Throughout the studies included in this review, prehabilitation interventions consistently did not lead to worsened outcomes and displayed promising implications for supporting post-operative outcomes, particularly in the domains of fitness and physical function. These findings underscore the value of a holistic approach to patient care that encompasses not only surgical and medical interventions but also proactive measures to optimize patient health before surgery. The potential benefits of pre-operative prehabilitation interventions in HPB cancer populations are promising. Integrating exercise and nutrition prehabilitation programs into clinical care should be considered a proactive step towards improving patient outcomes and enhancing overall quality of life in the face of these challenging cancers.

## Figures and Tables

**Table 1:** Overview of exercise-only pre-operative prehabilitation studies in patients with HPB cancers.

Author, Year, Country	Design and Groups	Cancer Type	Cancer Treatments	Sample (*n*)	Exercise Type/Setting	Intervention Details	Length (Weeks)	Frequency (Times per Week)	Intensity	Time (min per Session)
Dunne et al., 2016, United Kingdom [54]	RCT (EX vs. SOC)	Colorectal liver metastasis	Mixed	38	AE/Supervised	AE: HIIT (Cycling)	4	3	AE: <60% VO_2_max (low intervals) to 90% VO_2_max (high intervals)	30
Kitahata et al., 2018, Germany [45]	Retrospective cohort (EX vs. SOC)	Pancreatic, bile duct, duodenum, and other undergoing pancreato-duodenectomy	Mixed	576	COMB (AE + RE)/Mixed	AE: Stationary bike, treadmill, and stair stepping RE: NR Other: Breathing exercises	1	14 (twice a day)	AE: 60%VO_2_max RE: NR	70
Mikami et al., 2020, Japan [46]	Single group	Pancreatic	Mixed (*n* = 4 on preoperative chemotherapy)	26	COMB (AE + RE)/Supervised	AE: Cycle ergometer, handgrip ergometry, treadmill walking RE: Body weight	Varied 11.9 days (Median)	7 (once a day)	AE: 11–13 RPE (Borg scale) RE: NR	70
Ngo-Huang et al., 2017 and 2019 [47,48] Parker et al., 2019 and 2021, United States [49,50]	Single group	Pancreatic	Chemotherapy and/or chemoradiation	20	COMB (AE + RE)/ Home-based	AE: Walking RE: Full body (resistance bands, dumbbells, or machines)	Varied	2 to 3	AE: 12–13 RPE (Borg scale) RE: 12–13 RPE (Borg scale)	AE: ≥20 RE: ≥30

Abbreviations (Alphabetical): AE: Aerobic Exercise; EX: Exercise; COMB: Combined Intervention (AE + RE); HIIT: High-Intensity Interval Training; HPB: Hepato-Pancreato-Biliary; Min: Minute; NR: Not Reported; RCT: Randomized Clinical Trial; RE: Resistance Exercise; SOC: Standard of Care.

**Table 2:** Overview of multimodal pre-operative prehabilitation studies in patients with HPB cancers.

Author, Year, Country	Design and Groups	Cancer Type	Cancer Treatments	Sample (n)	Exercise Type/ Setting	Intervention Details	Length (Weeks)	Frequency (Times per Week)	Intensity	Time (min per Session)
Ausania et al., 2019, Spain [51]	RCT (EX vs. SOC)	Pancreatic or peripancreatic malignancy	No treatment	40	COMB (AE + RE)/ Mixed	AE: HIIT (supervised cycling and unsupervised functional exercises) RE: Home-based, NR NUT: Nutrition support (oral supplementation), endocrine and exocrine support	Varied 12.6 days (Median)	Supervised: 5 Home-based: Daily (following supervised period)	Supervised: High intensity Home-based: NR	Supervised: 60 total (20 AE + 20 RE) Home-based: NR
Baimas-George et al., 2020, United States [55]	Single group	HPB malignancy	Neoadjuvant chemotherapy	19	AE/Home-based	AE: NR NUT: NR Other: Psychological services)	16	NR	NR	NR
Bui-Ngoc et al., 2019, Canada [58]	RCT (Prehab vs. Rehab)	HPB and pancreatic	Neoadjuvant chemotherapy and no treatment	35	COMB (AE + RE)/Mixed	AE: Walking RE: Full body strength with body weight and resistance bands NUT: Whey protein powder Other: Relaxation for stress, anxiety, and pain management + targeted stretching	4	AE: Daily RE: 3–4	AE: 3–4 RPE (Borg scale) RE: 2 sets of 8–15 reps	AE: 30 RE: 30
Kaibori et al., 2013, Japan [53]	RCT (EX + Diet vs. Diet-alone)	Hepatocellular carcinoma	NR	51	AE/Supervised	AE: Walking (+targeted stretching) NUT: Adapted to either chronic hepatitis/liver cirrhosis or diabetes/fatty liver	4 (pre-operatively) + 1-week post-operative for 6 months	3	AE: Anaerobic threshold	60
Nakajima et al., 2019, Japan [56]	Non-randomized trial (EX vs. Historical Controls on SOC)	HPB malignancy	No treatment	142	COMB (AE + RE)/Home-based	AE: Walking RE: Body weight or free weights (squats, calf raises, sit-ups, bridge up, and upper limb) NUT: Leucine-rich amino acid supplement	Varied	3 (minimum)	AE: 3–4 RPE (Borg scale) RE: NR	30
Van Wijk et al., 2022, Netherlands [52]	Single group	Liver or pancreatic	NR	26	AE/Mixed	AE: Personalized cycle ergometer (2 HIIT and 1 MIIT sessions) NUT: Protein and multivitamin supplementation	4	AE: HIIT: 2 days/week AE: MIIT: 1 days/week	AE: HIIT: 60% of peak work rate to 20% of peak work rate AE: MIIT: 40% of peak work rate to 20% of peak work rate	30
Wang et al., 2020, Singapore [57]	Non-randomized trial (EX vs. Controls on SOC)	HPB	NR	104	COMB (AE + RE)/Home-based	AE: Walking RE: Individualized lower limb strengthening NUT: Tailored Other: Psychosocial care	2 to 4	5	NR	30

Abbreviations (Alphabetical): AE: Aerobic Exercise; EX: Exercise; COMB: Combined Intervention (AE + RE); HIIT: High-Intensity Interval Training; HPB: Hepato-Pancreato-Biliary; MIIT: Moderate-Intensity Interval Training; min: Minute; NR: Not Reported; NUT: Nutrition; RCT: Randomized Clinical Trial; RE: Resistance Exercise; SOC: Standard of Care.

**Table 3:** Summary of fitness, strength, and physical function outcomes after a prehabilitation intervention in patients with HPB cancers.

Study	Intervention	Main Result
Dunne et al., 2016, United Kingdom [54]	AE	⇑ VO_2_ peak (EX vs. SOC) ⇑ Peak work rate (EX vs. SOC) ↔ Heart rate reserve (EX vs. SOC)
Mikami et al., 2020, Japan [46]	COMB	⇑ VO_2_ peak (Pre vs. Post) ⇑ Peak work rate (Pre vs. Post) ⇑ 6MWT distance (Pre vs. Post)
Ngo-Huang et al., 2017, United States [48]	COMB	⇑ Grip strength (Pre vs. Post) ⇑ MET min/week of physical activity (Pre vs. Post)
Ausania et al., 2019, Spain [51]	COMB + NUT	⇑ Cardiopulmonary status (Pre vs. Post) ⇓ Time to complete 10 m walk test (Pre vs. Post) ⇑ Grip strength (Pre vs. Post)
Kaibori et al., 2013, Japan [53]	AE + NUT	↔ Muscle strength (Pre vs. Post) ⇓ Heart rate variability (Pre vs. Post)
Nakajima et al., 2019, Japan [56]	COMB + NUT	⇑ VO_2_ peak (Pre vs. Post) ⇑ Peak work rate (Pre vs. Post)
Ngo-Huang et al., 2019, United States [47]	COMB	⇑ 6MWT distance (Pre vs. Post) ⇓ Sit-to-stand time to completion (Pre vs. Post) ↔ Grip strength (Pre vs. Post) ⇑ Gait speed (Pre vs. Post)
Baimas-George et al., 2020, United States [55]	AE + NUT	↔ Grip strength (Pre vs. Post) ⇓ Frailty (Pre vs. Post) Frail phenotype Frailty-related weight loss Frailty-related exhaustion ↔ Timed-up-and-go (Pre vs. Post)
Nakajima et al., 2019, Japan [56]	COMB + NUT	⇑ 6MWT distance (Pre vs. Post) ↔ Gait speed (Pre vs. Post) ↔ Grip strength (Pre vs. Post)
Bui-Ngoc et al., 2019, Canada [58]	COMB + NUT	↔ 6MWT distance (Prehab: Baseline vs. Post) ⇓ 6MWT distance (Prehab: Pre vs. Post) ⇓ 6MWT distance (Rehab: Baseline vs. Post) ⇓ 6MWT distance (Rehab: Pre vs. Post) ↔ Grip strength (Prehab: Pre vs. Post) ↔ Grip strength (Rehab: Pre vs. Post) ↔ Arm curl—left and right side(Prehab: Pre vs. Post) ⇓ Arm curl—left side (Rehab: Pre vs. Post) ⇑ Timed-up-and-go (Prehab: Pre vs. Post) ↔ Timed-up-and-go (Rehab: Pre vs. Post) ⇓ Knee extension (Prehab: Pre vs. Post) ↔ Knee extension (Rehab: Pre vs. Post) ↔ Knee flexion (Prehab: Pre vs. Post) ↔ Knee flexion (Rehab: Pre vs. Post)

Footnotes: ⇑ or ⇓ = Significant change at *p* ≤ 0.05; ↔ = No significant difference at *p* > 0.05. Abbreviations (Alphabetical): AE: Aerobic Exercise; COMB: Combined Aerobic and Resistance; EX: Exercise; MET: Metabolic Equivalent; min: Minute; NUT: Nutrition;; SOC: Standard of Care; 6MWT: 6 min Walk Test.

**Table 4:** Summary of biomarker and nutritional status outcomes after a prehabilitation intervention in patients with HPB cancers.

Study	Intervention	Main Result
Baimas-George et al., 2020, United States [55]	AE + NUT	↔ Nutritional Status (Pre vs. Post) ↔ Glucose (Pre vs. Post) ↔ TSH (Pre vs. Post) ↔ Creatinine (Pre vs. Post)
Kaibori et al., 2013, Japan [53]	AE + NUT	↔ Serum albumin at 6 months post-operation (EX + Diet vs. Diet-Alone) ↔ Triglyceride levels at 6 months post-operation (EX + Diet vs. Diet-Alone) ↔ Cholesterol levels at 6 months post-operation (EX + Diet vs. Diet-Alone) ↔ Glucose levels at 6 months post-operation (EX + Diet vs. Diet-Alone) ↔ Fasting serum insulin and insulin resistance at post-operation (EX + Diet vs. Diet-Alone) ⇓ Fasting serum insulin and insulin resistance at 3 months and 6 months post-operation (EX + Diet vs. Diet-Alone)
Nakajima et al., 2019, Japan [56]	COMB + NUT	⇑ Prognostic nutritional index (EX vs. SOC) ⇑ Serum albumin levels (EX: vs. SOC)
Wang et al., 2020, Singapore [57]	COMB + NUT	↔ Serum albumin (EX vs. SOC)

Footnotes: ⇑ or ⇓ = Significant change at *p* ≤ 0.05; ↔ = No significant difference at *p* > 0.05. Abbreviations (Alphabetical): AE: Aerobic Exercise; COMB: Combined Aerobic and Resistance; EX: Exercise;; NUT: Nutrition; SOC: Standard of Care; TSH: Thyroid-Stimulating Hormone.

**Table 5:** Summary of body composition outcomes after a prehabilitation intervention in patients with HPB cancers.

Study	Intervention	Main Result
Parker et al., 2019 and Parker et al., 2021, United States [49,50]	COMB	↔ BMI (EX vs. SOC) ⇑ Skeletal muscle index (EX vs. SOC) ↔ Skeletal muscle density (EX vs. SOC) ⇓ BMI (EX: Pre vs. Post) ↔ Skeletal muscle index (EX: Pre vs. Post) ⇓ Skeletal muscle index (SOC: Pre vs. Post)
Kaibori et al., 2013, Japan [53]	AE + NUT	⇓ Whole body mass at 6 months post-operation (EX + Diet vs. Diet-Alone) ⇓ Body mass at waist and fat mass at waist at 6 months post-operation (EX + Diet vs. Diet-Alone) ↔ Whole body fat mass, fat-free mass, and bone mineral density at 6 months post-operation (EX + Diet vs. Diet-Alone)
Nakajima et al., 2019, Japan [56]	COMB + NUT	⇓ Body weight (EX: Within-group change; SOC: Within-group change) ⇓ BMI (EX: Within-group change; SOC: Within-group change) ↔ Skeletal muscle mass (EX: Within-group change) ⇓ Fat mass (EX: Within-group change) ⇑ Muscle-to-fat ratio (EX: Within-group change)
Wang et al., 2020, Singapore [57]	COMB + NUT	↔ BMI (EX vs. SOC)
Bui-Ngoc et al., 2019, Canada [58]	COMB + NUT	↔ Appendicular skeletal muscle index (Prehab: Pre vs. Post) ⇓ Appendicular skeletal muscle index (Rehab: Pre vs. Post)

Footnotes: ⇑ or ⇓ = Significant change at *p* ≤ 0.05; ↔ = No significant difference at *p* > 0.05. Abbreviations (Alphabetical): AE: Aerobic Exercise; BMI: Body Mass Index; COMB: Combined Aerobic and Resistance; EX: Exercise; NUT: Nutrition; SOC: Standard of Care.

**Table 6:** Summary of post-operative clinical outcomes after a prehabilitation intervention in patients with HPB cancers.

Study	Intervention	Main Result
Dunne et al., 2016, United Kingdom [54]	AE	↔ Readmission (EX vs. SOC) ↔ Bile duct reconstruction (EX vs. SOC) ↔ LOS (EX vs. SOC)
Kitahata et al., 2018, Germany [45]	COMB	⇓ Post-operative LOS (EX vs. SOC) ⇓ Pulmonary complications (EX vs. SOC) ↔ Severe complications (EX vs. SOC) ↔ Morbidity (EX vs. SOC) ↔ Mortality (EX vs. SOC) ↔ Bile leakage (EX vs. SOC) ↔ Delayed gastric emptying (EX vs. SOC)
Ausania et al., 2019, Spain [51]	COMB + NUT	↔ Complications (EX vs. SOC) ↔ Post-operative LOS (EX vs. SOC) ↔ Readmission (EX vs. SOC) ⇓ Delayed gastric emptying (EX vs. SOC)
Baimas-George et al., 2020, United States [55]	AE + NUT	↔ LOS (EX + Diet vs. Diet-Alone) ↔ Mortality (EX + Diet vs. Diet-Alone) ↔ Morbidity (EX + Diet vs. Diet-Alone) ↔ Post-operative blood loss (EX + Diet vs. Diet-Alone)
Van Wijk et al., 2022, Netherlands [52]	AE	⇓ Post-operative complication rate (EX vs. SOC) ⇓ Post-operative LOS (EX vs. SOC) ↔ Re-admission rate (EX vs. SOC)
Bui-Ngoc et al., 2019, Canada [58]	COMB + NUT	↔ Post-operative LOS (Prehab vs. Rehab) ↔ Rehospitalization (Prehab vs. Rehab)

Footnotes: ⇑ or ⇓ = Significant change at *p* ≤ 0.05; ↔ = No significant difference at *p* > 0.05. Abbreviations (Alphabetical)*:* AE: Aerobic Exercise; COMB: Combined Aerobic and Resistance; EX: Exercise; LOS: Length-of-Stay; NUT: Nutrition; SOC: Standard of Care.

**Table 7:** Ongoing clinical trials with exercise or exercise and nutrition prehabilitation interventions in patients with HPB cancers (http://ClinicalTrials.gov, assessed on 24 September 2023).

Identifier	Study Design	Population and Treatment Status	Experimental Groups	Intervention Characteristics	Outcomes of Interest
NCT05356117 [93]	RCT Length: 16 weeks	Pancreatic cancer on neoadjuvant chemotherapy aged 18 years and older	(1) Prehabilitation (RE + NUT) (2) Prehabilitation (RE Only) (3) Attention control	(1) RE + NUT: Home-based RE program, 3 days/week with daily protein supplementation. (2) RE Only: Home-based RE program, 3 days/week	Primary: Feasibility Secondary: Skeletal muscle mass, tissue wasting, physical function, muscular strength, physical fitness, patient-reported outcomes
NCT03475966 [103]	RCT Length: NR	Pancreatic cancer, liver cancer, bile duct cancer, aged 18–95 years old	(1) Prehabilitation (COMB, NUT, Other) (2) Rehabilitation	(1) Prehabilitation: Home-based COMB exercise program, as well as NUT with a high-protein diet, protein supplementation, and relaxation techniques. (2) Rehabilitation: Same components as the prehabilitation group, provided following surgery.	Primary: Physical function Secondary: Physical function, strength, body composition, nutritional status, post-operative complications, patient-reported outcomes, physical activity levels
NCT04602026 [94]	RCT Length: NR	Pancreatic, liver, or gastric cancer, aged 18 years and older	(1) Prehabilitation (EX) (2) SOC	(1) EX: Home-based EX program, including physical therapy	Primary: Frailty, post-operative complications, patient-reported outcomes
NCT05921552 [89]	Single group Length: Up to 4 weeks before surgery	Hepatocellular carcinoma or liver metastasis, aged 70 years and older	(1) Prehabilitation (COMB)	(1) COMB: Tele-health exercise program with RE 30 min/session, 2 days/week using resistance bands and AE for 30 min/session for at least 3 sessions per week at 50–70% of age-predicted maximum heart rate.	Primary: Feasibility, adherence, patient-reported outcomes, physical function, fitness, body composition
NCT05281211 [90]	RCT Length: 6 weeks	Liver cancer, aged 18–90 years with sarcopenia	(1) Prehabilitation (AE + NUT) (2) No intervention	(1) Prehabilitation: Walk 30 min/day (or 2000 extra steps) 6 weeks prior to surgery, with BCAA supplementation and immune-system boosters 2 times/day for 4 weeks and once daily for 2 weeks.	Primary: Post-operative morbidity Secondary: Post-operative complications, body composition
NCT05225038 [91]	RCT Length: At least 2 weeks before surgery	Pancreatic cancer receiving upfront surgery or neoadjuvant chemotherapy, aged 18 and older	(1) Prehabilitation (EX + NUT + Other) (2) SOC	(1) Prehabilitation: EX, NUT, and behavioral support (relaxation and smoking cessation)	Primary: Physical function Secondary: Post-operative outcomes, physical function, fitness
NCT04246970 [92]	RCT Length: 8 weeks before surgery	Liver cancer and other candidates for liver transplants, aged 18 years and older	(1) Prehabilitation (COMB) (2) Prehabilitation (COMB) + Rehabilitation (3) SOC	(1) Prehabilitation: COMB with AE done in 5 cycles of intervals of 2 min at 70% Watts or HR and 3 min of active rest and RE in 1–3 sets with 10–15 reps per exercise at 5–6 out of 10 on Borg RPE scale. Additionally inspiratory muscle training and balance training. (2) Prehabilitation + Rehabilitation: Same structure as prehabilitation program with a 12-week rehabilitation program consisting of a supervised and unsupervised COMB program 5 days/week.	Primary: Post-operative complications, physical function Secondary: Cardiovascular fitness, physical function, strength, body composition, patient-reported outcomes
NCT05489419 [96]	Single group Length: 3–4 weeks	Pancreatic cancer, any ages	(1) Prehabilitation (AE + NUT)	(1) AE: Through physical and cardiopulmonary training + personalized nutrition, as well as anxiety and depression treatment.	Primary: Cardiovascular fitness and aerobic capacity Secondary: Adherence, body composition, biomarkers of inflammation, patient-reported outcomes, post-operative complications
NCT03865875 [102]	Single group Length: NR	Pancreatic cancer, aged 18 years and older	(1) Prehabilitation (COMB + NUT)	(1) Prehabilitation: Standardized exercise program and individualized nutrition plan.	Primary: Completion of program
NCT05836870 [100]	RCT Length: NR	Pancreatic cancer, receiving neoadjuvant chemotherapy, aged 18 years and older	(1) Prehabilitation (RE) (2) Enhanced usual care	(1) RE: Tele-supervised RE program. (2) Enhanced usual care: Provided recommendations about physical activity and nutrition.	Primary: Physical function and post-operative chemotherapy initiation Secondary: Patient-reported outcomes, post-operative outcomes, body composition, fitness, physical function, nutrition, biomarkers
NCT05483075 [101]	Non-randomized trial Length: 2–4 weeks	Pancreatic cancer, aged 18–90 years	(1) Prehabilitation (COMB) (2) SOC	(1) Prehabilitation: Supervised moderate-intensity COMB exercise lead by a health care provider at least AE 5 times/week for 30 min/session and RE for 2 days/week.	Primary: Compliance Secondary: Post-operative outcomes, biomarkers, fitness
NCT04469504 [95]	RCT Length: 4 weeks	Pancreatic cancer patients with sarcopenia, aged 18 years and older	(1) Prehabilitation: EX, NUT, Other (2) Control: NUT	(1) Prehabilitation: EX, NUT supplementation, and psychological support (2) NUT Control: Perioperative immunonutrition	Primary: Post-operative outcomes Secondary: Post-operative outcomes, patient-reported outcomes
NCT05044065 [99]	RCT Length: 5–6 weeks (both pre- and post-operative)	Pancreatic and biliary cancer, aged 18 years and older	(1) Prehabilitation and Rehabilitation: RE and NUT (2) Control: NUT	(1) Prehabilitation and Rehabilitation: Pre-operative prehabilitation and post-operative rehabilitation with supervised and home-based RE with NUT protein supplementation (2) NUT Control: NUT protein supplementation	Primary: Body composition Secondary: Nutritional biomarkers, body composition, strength, patient-reported outcomes, fitness, post-operative outcomes, biomarkers
NCT04923672 [97]	RCT Length: At least 3 weeks before surgery	Hepatobiliary cancer or colorectal cancer, aged 18 years and older	(1) Prehabilitation: Moderate AE (2) Prehabilitation: High-intensity interval training (HIIT) AE (3) Control	(1) Prehabilitation: Moderate AE: Increase activity to 5 days/week, 40 min/day (2) Prehabilitation: HIIT AE: 5 days/week 40 min/day of moderate and vigorous activity	Primary: Steps per day Secondary: Compliance, patient-reported outcomes, post-operative outcomes, fitness
NCT03187951 [98]	RCT Length: During chemotherapy and up to 7 months post-operation	Pancreatic cancer scheduled to receive neoadjuvant chemotherapy and/or radiation before surgery, aged 18 years and older	(1) Prehabilitation and Rehabilitation: COMB + NUT (2) Control: NUT	(1) Prehabilitation and Rehabilitation: Moderate-intensity AE for at least 5 days/week, 30 min/session, and RE using resistance bands at least 2 days/week for 8 exercises at 1 set, 10–15 reps, with progressive increase in resistance. NUT counseling provided. (2) NUT Control: Stretching program and nutritional counseling provided.	Primary: Fitness

Abbreviations (Alphabetical): AE: Aerobic Exercise; BCAA: Branched-Chain Amino Acid; COMB: Combined; EX: Exercise; HIIT: High-Intensity Interval Training; HPB: Hepato-Pancreato-Biliary; HR: Heart Rate; min: Minute; NR: Not Reported; NUT: Nutrition; RCT: Randomized Clinical Trial; RE: Resistance Exercise; reps: Repetitions; RPE: Rating of Perceived Exertion.

## Data Availability

No new data were created or analyzed in this study. Data sharing is not applicable to this article.
